# The Molecular Basis for Altered Cation Permeability in Hereditary Stomatocytic Human Red Blood Cells

**DOI:** 10.3389/fphys.2018.00367

**Published:** 2018-04-16

**Authors:** Joanna F. Flatt, Lesley J. Bruce

**Affiliations:** Bristol Institute for Transfusion Sciences, NHS Blood and Transplant, Bristol, United Kingdom

**Keywords:** hereditary stomatocytosis, familial pseudohyperkalemia, SLC4A1, SLC2A1, RhAG, ABCB6, PIEZO1, KCNN4

## Abstract

Normal human RBCs have a very low basal permeability (leak) to cations, which is continuously corrected by the Na,K-ATPase. The leak is temperature-dependent, and this temperature dependence has been evaluated in the presence of inhibitors to exclude the activity of the Na,K-ATPase and NaK2Cl transporter. The severity of the RBC cation leak is altered in various conditions, most notably the hereditary stomatocytosis group of conditions. Pedigrees within this group have been classified into distinct phenotypes according to various factors, including the severity and temperature-dependence of the cation leak. As recent breakthroughs have provided more information regarding the molecular basis of hereditary stomatocytosis, it has become clear that these phenotypes elegantly segregate with distinct genetic backgrounds. The cryohydrocytosis phenotype, including South-east Asian Ovalocytosis, results from mutations in *SLC4A1*, and the very rare condition, stomatin-deficient cryohydrocytosis, is caused by mutations in *SLC2A1*. Mutations in *RHAG* cause the very leaky condition over-hydrated stomatocytosis, and mutations in *ABCB6* result in familial pseudohyperkalemia. All of the above are large multi-spanning membrane proteins and the mutations may either modify the structure of these proteins, resulting in formation of a cation pore, or otherwise disrupt the membrane to allow unregulated cation movement across the membrane. More recently mutations have been found in two RBC cation channels, *PIEZO1* and *KCNN4*, which result in dehydrated stomatocytosis. These mutations alter the activation and deactivation kinetics of these channels, leading to increased opening and allowing greater cation fluxes than in wild type.

## Introduction

At first glance, mature human red blood cells (RBCs) appear to be simply membranes packed full of hemoglobin, carrying the respiratory gases oxygen and carbon dioxide around the body. They lack all cellular organelles, even the seemingly indispensible nucleus and mitochondria, enabling the maximum volume of the cells to be occupied by hemoglobin. Consequently, RBCs contain extremely high concentrations of hemoglobin (30–35 g/dL) and other impermeant solutes, creating an osmotic imbalance between the intracellular space and the surrounding plasma. Upon closer inspection, the RBC membrane has a greater role to play in RBC function than solely to contain the large amounts of hemoglobin required for oxygen transport. Interactions between RBC membrane proteins and the underlying cytoskeleton maintain the RBC's unique biconcave discoid shape that enables the 6 μm cell to deform and pass through 3 μm capillaries and splenic slits. Some of the most abundant RBC membrane proteins are fundamental to the rapid and efficient transport gases, for example Rh-associated glycoprotein (RhAG) and aquaporin 1 (AQP1) transport neutral gases such as carbon dioxide (Endeward et al., 2006) and maybe oxygen and/or nitric oxide (Burton and Anstee, 2008). The glucose transporter 1 (GLUT1) transports essential metabolites such as glucose (for energy) and dehydroascorbic acid (DHA; for redox homeostasis). The most abundant RBC membrane protein, band 3, exchanges bicarbonate to the plasma in exchange for chloride, a process that is crucial for the efficient transport of carbon dioxide in the blood. This high membrane permeability to anions is in contrast to its very low permeability to cations. In human RBCs it is thought that the cell utilizes its low cation permeability to counteract the osmotic effect of the high solute concentration within the RBCs. Cation gradients across the membrane are actively maintained by pumps keeping intracellular sodium low and intracellular potassium high. This mechanism is tightly regulated to ensure that there is no uncontrolled movement of water into or out of the RBC and is fundamental to cellular volume control (reviewed in Gallagher, 2017).

## Cation pumps, transporters and channels in the RBC membrane

Human RBC membranes contain a number of different cation pumps, transporters and channels (Gallagher, 2017). The major protein responsible for maintaining the high potassium, low sodium intracellular state is the Na,K-ATPase, which is an ATP-dependent pump that exchanges 3 sodium ions outwards for 2 potassium ions inwards. Its activity can be specifically inhibited with ouabain. A second major physiologically-active transporter is the NaK2Cl cotransporter (NKCC1), which usually moves 1 Na^+^, 1 K^+^, and 2 Cl^−^ into the cell from the extracellular space, although transport can occur in both directions across the membrane. The NaK2Cl cotransporter is inhibited by the drugs bumetanide and furosemide, cell swelling and urea (Lim et al., 1995), and is expressed in numerous cell types. Another ion transporter present in the RBC membrane is the potassium chloride cotransporter (KCC3), which is stimulated by cell swelling and urea. This protein cotransports both potassium and chloride out of the cell, which are accompanied by water and leads to a decrease in cell volume (Lauf and Adragna, 2000). The Na-H exchanger (NHE1) is present in RBCs and many other cell types, and is involved in cell volume and pH control. It can be inhibited with the drug amiloride. The cytoplasmic domain of NHE1 interacts with the cytoskeletal protein 4.1, which may play a part in its regulation (Nunomura et al., 2012). It is activated by cell shrinking, which is associated with phosphorylation of a number of residues of NHE1 (Rigor et al., 2011). The non-selective, voltage-dependent cation (NSVDC) channel (Bennekou and Christopherson, 2003) and the K(Na)/H exchanger (Bernhardt and Weiss, 2003) are both activated in low ionic strength conditions and may play a role in cation loss in low chloride media, but probably have little role in cation homeostasis at physiological pH and tonicity.

Many of these transporters are regulated by changes in cell volume (swelling and/or shrinkage). The mechanisms underlying this are likely to involve protein-protein interactions, transporter phosphorylation, and cell chloride concentrations (Flatman, 2002). Other cation transporting proteins are not thought to play a major role within homeostatic physiological conditions. These include the calcium-activated potassium channel, KCNN4, known as KCa3.1 or the Gardos channel (Hoffman et al., 2003). Elevation of intracellular calcium levels activates the Gardos channel to its open conformation, allowing potassium efflux and consequent rapid cellular dehydration. However, this channel usually exists in its dormant closed state, as intracellular calcium is extruded by the Ca^2+^-ATPase. Although the RBC membrane has been very well studied, new insights continue to be made concerning the proteins present and their role in RBC function. There is some evidence from studies in mice that the Kell/XK complex is involved in the regulation of cation homeostasis, as the absence of XK leads to elevated intracellular calcium levels and Gardos channel activation (Rivera et al., 2013a).

Fairly recently it was discovered that the RBC membrane contains a mechanosensitive cation channel, PIEZO1 (Zarychanski et al., 2012). PIEZO1 is encoded by the gene *FAM38A* and can be inhibited by the stretch-activated channel inhibitor, tarantula toxin GsMTx4 (Bae et al., 2011). A synthetic small molecule called Yoda1 has been shown to act as a specific PIEZO1 agonist, and may prove to be an important tool for full investigation of the biophysical properties of the channel (Syeda et al., 2015). PIEZO1 has been knocked down in zebrafish and mouse, resulting in swelling and lysis of RBCs, suggesting that it is important for cell volume control (Faucherre et al., 2014; Cahalan et al., 2015), and it has been shown to be important for vascular development (Ranade et al., 2014). Evidence from a recent study by Cahalan et al. suggests that PIEZO1-mediated calcium influx activates the Gardos channel and triggers cellular dehydration. This mechanism has been hypothesized to aid the RBC's passage through the microvasculature by allowing it to reduce its volume in response to mechanical stimulation or shear stress (Cahalan et al., 2015). It will be interesting to discover whether PIEZO1 is directly involved in regulating other RBC membrane cation transporters that are variously stimulated or inhibited by cell swelling, raising the exciting possibility that PIEZO1 is the master regulator of RBC volume control.

## Normal membrane ion distribution and fluxes

**Figure 1:**
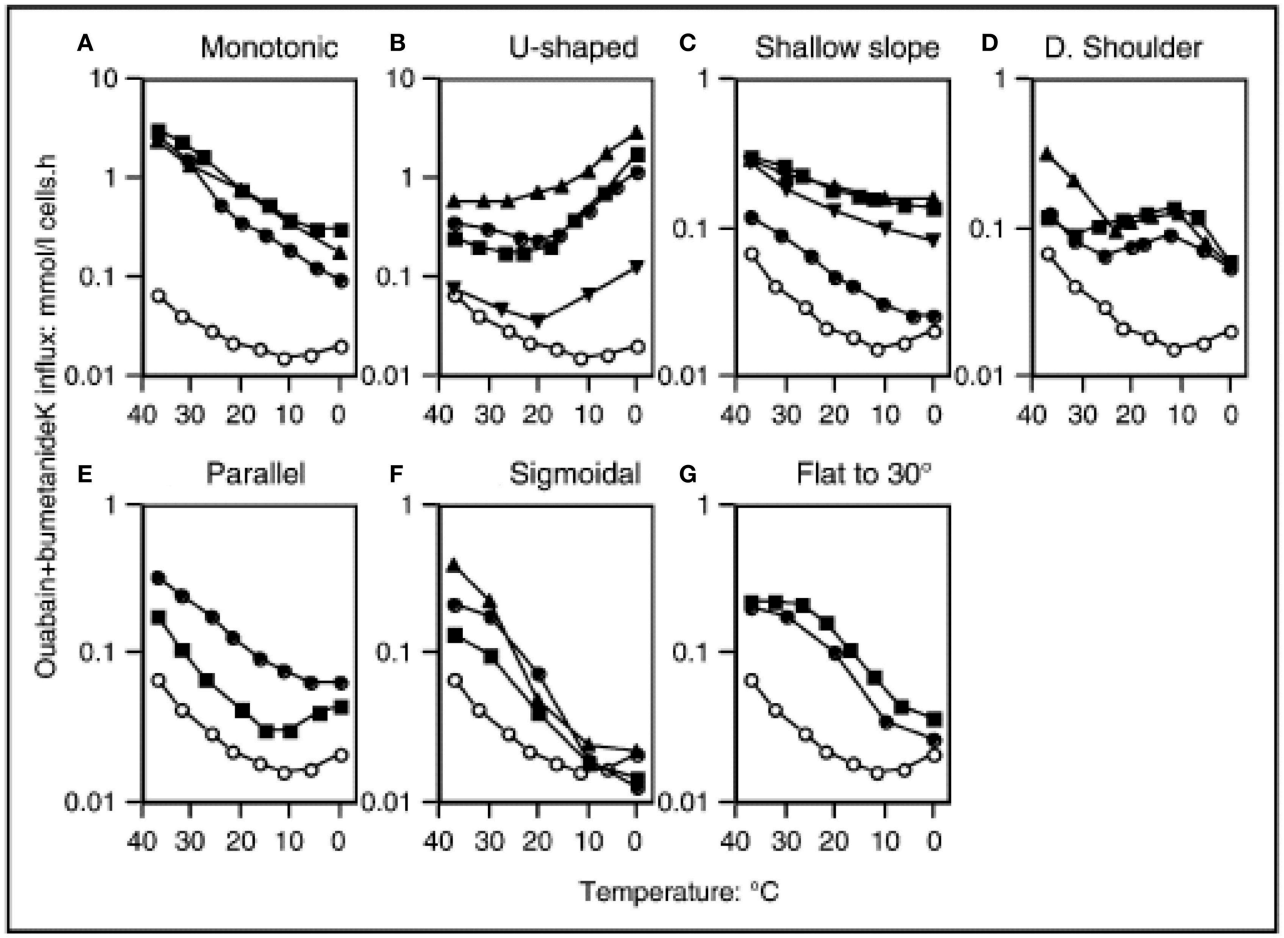
Temperature dependence of “leak” potassium flux in different leaky membrane variants among the hereditary stomatocytosis conditions. Open symbols denote normal red cells. Closed symbols denote patients, detailed below and in the Table. Potassium influx was measured using ^86^Rb as a tracer. The medium contained (mM): Na^+^, 145: K^+^, 5; Cl^−^, 150, MOPS, 15 (pH 7.4 at 20°C); glucose, 5; ouabain, 0.1; bumetanide, 0.1. Reproduced with permission from Stewart (2004).

In normal human RBCs there is a very low basal permeability to cations. At body temperature, this “leak” is corrected by the Na,K-ATPase, which is present in RBCs at a low copy number, but is sufficient to maintain the sodium/potassium electrochemical gradients. This basal cation permeability can be measured in the presence of the inhibitors ouabain and bumetanide, in order the exclude the activity of the Na,K-ATPase and NaK2Cl transporter, respectively. The temperature-dependence of the potassium leak has been evaluated, and it exhibits an unusual pattern. From 37°C downwards the leak decreases with temperature in a monotonic manner. The minimum of the leak occurs at around 8°C, but below 10°C the leak actually increases with decreasing temperature, resulting in a U-shaped graph (Figure 1) (Stewart et al., 1980; Coles and Stewart, 1999). The explanation for this pattern is not known, and comparisons with other species' RBCs revealed that human RBCs appear to be unique in this feature (Hall and Willis, 1986).

## Comparison of human with other species' RBCs

Intriguingly, this use of cation gradients for regulation of cell volume in human RBCs does not appear to be a common feature of other mammals' RBCs. Dogs, sheep and cows are all species in which individuals' RBC phenotype may be categorized as either “high K” or “low K” (Chan et al., 1964; Gibson, 2003). In dogs the “low K” phenotype is most common, and this is inherited in an autosomal dominant manner. Despite the fact that the potassium levels of “high K” dog RBCs are most similar to the normal state of human RBCs, they exhibit signs of overhydration, including increased osmotic fragility, increased cell size, reduced cell hemoglobin concentration and reduced RBC lifespan. Studies of dog RBCs suggest that they employ a different mechanism for regulating their volume, predominantly involving the Na/H exchanger and KCl cotransporter (Parker et al., 1990). “High K” dog RBCs have higher levels of the Na,K-ATPase, which is thought to be responsible for the unusual cation gradients, and in this species its expression appears to be detrimental. It has been shown that both “low K” and “high K” dog reticulocytes express Na,K-ATPase and produce exosomes containing the protein, but “high K” erythroid progenitor cells express higher levels (Komatsu et al., 2010). The genetic basis of the “high K” vs. “low K” phenotype has not yet been established, but may provide a clue concerning the regulation of Na,K-ATPase expression.

Studies using duck RBCs have shown that their volume regulation involves coordinated transport by NaK2Cl cotransporter and KCl cotransporter proteins (Lytle and McManus, 2002), highlighting that there are many ways to control RBC volume, utilizing different transporters to achieve this goal.

Mice are often used as a model for the human system, however evaluation of a number of different mouse strains has revealed that their cation content and transporter activity are different depending on the strain and gender of the mouse (Rivera et al., 2013b). These observations may be useful in the investigation of genetic determinants of ion transport.

## Conditions involving disturbances in ion fluxes

It has become apparent that a number of factors can affect the cation permeability of the human RBC membrane. These include the use of certain drugs and oxidation of membranes. A recent study using aquaporin-9 knock-out mouse RBCs showed that oxidative modification of aquaporin-9 increases the cation permeability of the RBC membrane (Kucherenko et al., 2012). The presence of certain disease states results in aberrant membrane ion flux. Increased cation permeability has been recorded in thalassemia and chronic dyserythropoietic anemia (CDA), where the bone marrow is under continual stress (Wiley, 1981). Cation fluxes are also disturbed in a group of inherited disorders collectively known as the hereditary stomatocytoses. Although a common theme running throughout these disorders is that cation permeability of the RBC membrane is perturbed, these disorders show wide heterogeneity in the severity and characteristics of their cation leaks and also accompanying symptoms (Table 1). These are an extensively-studied group whose molecular bases remained unknown for a long time, but for the vast majority these have now been elucidated and form the basis of this review.

**Table 1:** Hereditary stomatocytosis pedigrees and other cation-leaky membrane disorders.

**Clinical/hematology**								**Genetics/biochemistry**		
**Phenotype**	**Pedigree**	**RBC morphology**	**Thromb**.	**Lipid**	**Leak rate**	**Temp dependence**	**References**	**Gene**	**Genotype**	**References**
OHSt 1	Stockport	Stomatocytes	+	Normal	× 40	Monotonic	Lock et al., 1961	*RHAG*	Phe65Ser	Bruce et al., 2009
OHSt 1	Albuquerque	Stomatocytes	No splen.	Normal	× 40	Monotonic	Fricke et al., 2004	*RHAG*	Phe65Ser	Bruce et al., 2009
OHSt 1	Brighton	Stomatocytes (30–40%)	+		× 40	Monotonic	Meadow, 1967	*RHAG*	Phe65Ser	Bruce et al., 2009
OHSt 1	Toulouse	Stomatocytes	No splen.		× 40	Monotonic	Fricke et al., 2004	*RHAG*	Phe65Ser	Bruce et al., 2009
OHSt 1	Grenoble	Stomatocytes (15–20%)	No splen.		× 40	Monotonic	Morlé et al., 1989	*RHAG*	Phe65Ser	Bruce et al., 2009
OHSt 1	Nancy	Stomatocytes (10–20%)	Unknown		× 40	Monotonic	Bruce et al., 2009	*RHAG*	Phe65Ser	Bruce et al., 2009
OHSt 2	Harrow	Stomatocytes	No splen.		× 40	Monotonic	Fricke et al., 2004	*RHAG*	Ile61Arg	Bruce et al., 2009
sdCHC + neuro.	Montpellier	Stomatocytes	No splen.		× 10	U-shaped	Fricke et al., 2004	*SLC2A1*	Gly286Asp	Flatt et al., 2011
sdCHC + neuro.	San Francisco	Unknown	No splen.		× 10	U-shaped	Fricke et al., 2004	*SLC2A1*	ΔIle435/436	Flatt et al., 2011
sdCHC + neuro.	n/a	Echinocytes, stomatocytes	No splen.			n.t.	Bawazir et al., 2012	*SLC2A1*	ΔIle435/436	Bawazir et al., 2012
PED + cation leak	n/a	Echinocytes (14–40%)	No splen.			n.t.	Weber et al., 2008	*SLC2A1*	Δ282–285	Weber et al., 2008
CHC	Hemel	Stomatocytes (30%)	−	+ ether	× 4	U-shaped	Coles et al., 1999a	*SLC4A1*	Ser731Pro	Bruce et al., 2005
CHC	Watford	Stomatocytes	−	+ ether	× 4	U-shaped	Coles et al., 1999a	*SLC4A1*	Ser731Pro	Bruce et al., 2005
CHC	Bushey	Stomatocytes	−	+ ether	× 4	U-shaped	Haines et al., 2001a	*SLC4A1*	Ser731Pro	Bruce et al., 2005
CHC	Milton Keynes		No splen.		× 4	U-shaped	Stewart, 2004	*SLC4A1*	Ser731Pro	Bruce et al., 2005
CHC	Hurstpierpoint	Stomatocytes (15–20%)	−	Normal	× 4	U-shaped	Haines et al., 2001a	*SLC4A1*	His734Arg	Bruce et al., 2005
CHC	Zurich		No splen.		× 4	U-shaped	Stewart, 2004	*SLC4A1*	His734Arg	Bruce et al., 2005
CHC	Islington					n.t.	Stewart, 2004	n.t.		
CHC	n/a	Stomatocytes, spherocytes			× 3	U-shaped	Guizouarn et al., 2011	*SLC4A1*	Ser762Arg	Guizouarn et al., 2011
SAO	n/a	Stomatocytic ovalocytes			× 2	U-shaped	Guizouarn et al., 2011	*SLC4A1*	Δ400–408	Tanner et al., 1991
HS-LTL	Cricklewood	Spherocytes	No splen.				Bruce et al., 2005	*SLC4A1*	Arg760Gln	Bruce et al., 2005
HS-LTL	Dagenham	Spherocytes	No splen.				Bruce et al., 2005	*SLC4A1*	Arg760Gln	Bruce et al., 2005
HS-LTL	Eastbourne	Spherocytes	+				Gore et al., 2002a	*SLC4A1*	Asp705Tyr	Bruce et al., 2005
HSt Blackburn	Blackburn	Stomatocytes	+	Normal	× 4	Shallow slope	Coles et al., 1999b	*SLC4A1*	Leu687Pro	Bruce et al., 2005
HSt Blackburn	Darlington	Stomatocytes (5–10%)	+		× 3	Shallow slope	Gore et al., 2004	*SLC4A1*	Leu687Pro	Bruce et al., 2005
HSt Blackburn	Bergen		+		× 4	Shallow slope	Stewart, 2004	n.t.		
HSt Blackburn	St. Etienne		+		× 4	Shallow slope	Stewart, 2004	n.t.		
Band 3 New Haven	Patient 1	Rare stomatocytes					Stewart et al., 2010	*SLC4A1*	Glu758Lys	Stewart et al., 2010
Band 3 New Haven	Patient 2	Mild anisocytosis					Stewart et al., 2010	*SLC4A1*	Glu758Lys	Stewart et al., 2010
HSt variant	Stanford	Normal, rare stomatocytes	Unknown		× 6		Stewart et al., 2011	*SLC4A1*	Arg730Cys	Stewart et al., 2011
Band 3 Ceinge + dyseryth.	Ceinge	Anisopoikilocytosis					Iolascon et al., 2009	*SLC4A1*	Gly796Arg	Iolascon et al., 2009
FP	Cardiff	Normal	No splen.	+ ether	× 1	U-shaped	Gore et al., 2002b	*ABCB6*	Arg723Gln	Bawazir et al., 2014
FP	Cumbria	Normal			× 1	U-shaped	Bawazir et al., 2014	*ABCB6*	Arg723Gln	Bawazir et al., 2014
FP	Harrow	Normal			× 1	U-shaped	Bawazir et al., 2014	*ABCB6*	Arg723Gln	Bawazir et al., 2014
FP	Cardiff-2						Andolfo et al., 2016	*ABCB6*	Arg723Gln /Arg276Trp	Andolfo et al., 2016
FP	Bolivian						Andolfo et al., 2016	*ABCB6*	Val454Ala	Andolfo et al., 2016
FP	Lille						Dagher et al., 1989	*ABCB6*	Arg375Gln	Andolfo et al., 2013a
FP	Chiswick	Normal	No splen.	Normal	× 1.5	Shoulder	Haines et al., 2001b	*ABCB6*	Arg375Gln	Bawazir et al., 2014
FP	White City						Bawazir et al., 2014	*ABCB6*	Arg375Gln	Bawazir et al., 2014
FP	Falkirk		No splen.	Normal	× 1.5	Shoulder	Haines et al., 2001a	*ABCB6*	Arg375Trp	Andolfo et al., 2013a
FP	Bow	Normal, rare stomatocytes	No splen.	Normal	× 1.5	Shoulder	Gore et al., 2004	*ABCB6*	Arg375Trp	Andolfo et al., 2013a
FP/DHSt	Irish		+				Stewart et al., 1996	*ABCB6*	Arg276Trp	Andolfo et al., 2016
DHSt M'brough	Middlesbrough	Target cells, rare stomatocytes			× 4	Flat to 30	Gore et al., 2004			
DHSt M'brough	Birmingham	Target cells			× 2	Flat to 30	Gore et al., 2004			
HSt variant	Woking	Stomatocytes (5–10%)	No splen.		× 4	Parallel	Jarvis et al., 2001			
DHSt + ascites	Bury St. Edmunds				× 2	Sigmoidal	Stewart, 2004			
DHSt + ascites	Sunderland	Anisocytosis, target cells	No splen.		× 2	Sigmoidal	Basu et al., 2003			
DHSt	Kyoto	Rare stomatocytes		+ PC			Imashuku et al., 2016	*FAM38A*	Glu2496GluLeuGlu	Imashuku et al., 2016
DHSt	Case 1						Albuisson et al., 2013	*FAM38A*	Glu2496GluLeuGlu	Albuisson et al., 2013
DHSt	Case 2						Albuisson et al., 2013	*FAM38A*	Glu2496GluLeuGlu	Albuisson et al., 2013
DHSt	Case 3						Albuisson et al., 2013	*FAM38A*	Glu2496GluLeuGlu	Albuisson et al., 2013
DHSt	Case 5						Albuisson et al., 2013	*FAM38A*	Glu2496GluLeuGlu	Albuisson et al., 2013
DHSt	Case 6						Albuisson et al., 2013	*FAM38A*	Glu2496GluLeuGlu	Albuisson et al., 2013
DHSt	Case 7						Albuisson et al., 2013	*FAM38A*	Glu2496GluLeuGlu	Albuisson et al., 2013
DHSt + ascites	Vesoul		+		× 2		Grootenboer et al., 2000	*FAM38A*	Glu2496GluLeuGlu	Albuisson et al., 2013
DHSt + FP	Family 3		+	+PC			Carli et al., 2007	*FAM38A*	Glu2496GluLeuGlu	Albuisson et al., 2013
DHSt + ascites	Patient 6		No splen				Glogowska et al., 2017	*FAM38A*	Glu2496GluLeuGlu	Glogowska et al., 2017
DHSt	Patient 7		No splen				Glogowska et al., 2017	*FAM38A*	Glu2496GluLeuGlu	Glogowska et al., 2017
DHSt + ascites	Patient 8		+				Glogowska et al., 2017	*FAM38A*	Glu2496GluLeuGlu	Glogowska et al., 2017
DHSt	Patient 9		+				Glogowska et al., 2017	*FAM38A*	Glu2496GluLeuGlu	Glogowska et al., 2017
DHSt	Family A (UK)	Schistocytes, rare stomatocytes					Andolfo et al., 2018a	*FAM38A*	Glu2496GluLeuGlu	Andolfo et al., 2018a
DHSt	Family B (Italy)	Schistocytes, rare stomatocytes					Andolfo et al., 2018a	*FAM38A*	Glu2496GluLeuGlu/Arg1864His	Andolfo et al., 2018a
DHSt + FP	Arras		+				Grootenboer et al., 2000	*FAM38A*	Arg2488Gln /Gly718Ser	Andolfo et al., 2013b
DHSt	Boston	Target cells					Platt et al., 1981	*FAM38A*	Arg2488Gln	Archer et al., 2014
DHSt + ascites	Patient 3		No splen				Glogowska et al., 2017	*FAM38A*	Arg2488Gln	Glogowska et al., 2017
DHSt	Canada	Target cells, schistocytes					Houston et al., 2011	*FAM38A*	Arg2456His	Zarychanski et al., 2012 Bae et al., 2013
DHSt	San Francisco						Grootenboer et al., 2000	*FAM38A*	Arg2456His	Andolfo et al., 2013b; Bae et al., 2013
DHSt	Rosedale	Macrocytes, stomatocytes					Shmukler et al., 2014	*FAM38A*	Arg2456His	Shmukler et al., 2014
DHSt	Denmark	Normal					Sandberg et al., 2014	*FAM38A*	Arg2456His	Sandberg et al., 2014
DHSt + ascites	Nantes family 1	Target cells, rare stomatocytes					Beneteau et al., 2014	*FAM38A*	Arg2456His	Beneteau et al., 2014
DHSt + ascites	Nantes family 2	Target cells, rare stomatocytes					Beneteau et al., 2014	*FAM38A*	Arg2456His	Beneteau et al., 2014
DHSt	Burnley		No splen.	+ PC	× 2	Parallel	Stewart, 2004	*FAM38A*	Arg2456His	Unpublished, LJ Bruce 2018
DHSt	Nuneaton		+	+ PC	× 2	Parallel	Stewart, 2004	*FAM38A*	Arg2456His	Unpublished, LJ Bruce 2018
DHSt	Patient 4		No splen				Glogowska et al., 2017	*FAM38A*	Arg2302His	Glogowska et al., 2017
DHSt	New York	Stomatocytes (up to 35% hom)		Normal			Miller et al., 1971	*FAM38A*	Met2225Arg	Zarychanski et al., 2012; Bae et al., 2013
DHSt	Dax						Grootenboer et al., 2000	*FAM38A*	2166-2169delLys	Andolfo et al., 2013b
DHSt + FP	Edinburgh	Target cells	No splen.	Normal	× 1.1	Shallow slope	Stewart and Ellory, 1985	*FAM38A*	Thr2127Met	Andolfo et al., 2013b
DHSt	Case 8						Albuisson et al., 2013	*FAM38A*	Thr2127Met	Albuisson et al., 2013
DHSt	Patient 2		No splen				Glogowska et al., 2017	*FAM38A*	Arg2088Gly	Glogowska et al., 2017
DHSt + FP	Vanves	Rare stomatocytes			× 1.5		Grootenboer et al., 2000	*FAM38A*	Ala2020Thr	Albuisson et al., 2013
DHSt	Essex						Carella et al., 1998	*FAM38A*	Ala2020Val/Ser1117Leu	Andolfo et al., 2013b
DHSt		Stomatocytes					Paessler and Hartung, 2015	*FAM38A*	c.6239_6256dup18	Paessler and Hartung, 2015
DHSt	Troyes		+				Grootenboer et al., 2000	*FAM38A*	Ala2003Asp	Andolfo et al., 2013b
DHSt	Patient 1		No splen				Glogowska et al., 2017	*FAM38A*	Arg1943Gln	Glogowska et al., 2017
DHSt	Case 4	Rare stomatocytes					Syfuss et al., 2006	*FAM38A*	Arg1358Pro	Albuisson et al., 2013
DHSt + FP + ascites	Bicetre						Grootenboer et al., 2000	*FAM38A*	Arg808Gln/Gly782Ser	Andolfo et al., 2013b
DHSt	Italy	Stomatocytes	+				Fermo et al., 2017	*KCNN4*	Arg352His	Rapetti-Mauss et al., 2015
DHSt	France	Anisocytosis					Rapetti-Mauss et al., 2015	*KCNN4*	Arg352His	Rapetti-Mauss et al., 2015
DHSt	Poland	Anisocytosis					Rapetti-Mauss et al., 2015	*KCNN4*	Arg352His	Andolfo et al., 2015
DHSt	Naples	Anisopoikilocytosis, stomatocytes					Andolfo et al., 2015	*KCNN4*	Arg352His	Andolfo et al., 2015; Glogowska et al., 2015
DHSt	Worcester				× 3		Grootenboer et al., 2000	*KCNN4*	Val282Met	Glogowska et al., 2015
DHSt	French/Irish	Target cells, acanthocytes					Glader et al., 1974	*KCNN4*	Val282Glu	Fermo et al., 2017

## Hereditary stomatocytoses

Despite the lack of information concerning the molecular cause(s) of the hereditary stomatocytoses (HSt), great efforts were made to thoroughly characterize the cation leaks exhibited by the known pedigrees. These analyses further underlined the wide heterogeneity of the disorders. Prof. G. Stewart classified the pedigrees into distinct types according to various factors, including the severity and temperature-dependence of the cation leak (Stewart, 2004) (Table 1, Figure 1). As recent breakthroughs have provided more information regarding the molecular basis of HSt, it has become clear that these phenotypes elegantly segregate with distinct genetic backgrounds.

## HSt mutations in large multi-spanning membrane proteins

### Band 3 mutations

#### Cryohydrocytosis

Cryohydrocytosis (CHC) is so named because of the temperature-dependence of the cation leak. Although CHC cells have increased basal cation permeability at physiological temperatures, this leak becomes much more pronounced when the cells are cooled to below 10°C (Figure 1B). Cryohydrocytosis was the first of the hereditary stomatocytoses in which the genetic basis was established. Heterozygous single amino acid substitutions in the band 3 protein (SLC4A1) were identified in 11 stomatocytosis pedigrees (Bruce et al., 2005). Band 3 is responsible for anion exchange across the plasma membrane, however in each of these pedigrees the mutant band 3 proteins were unable to transport anions even when expressed in the RBC membrane. Instead, expression of the mutant protein resulted in the temperature-sensitive increase in the permeability of the RBC membrane to cations.

**Figure 2:**
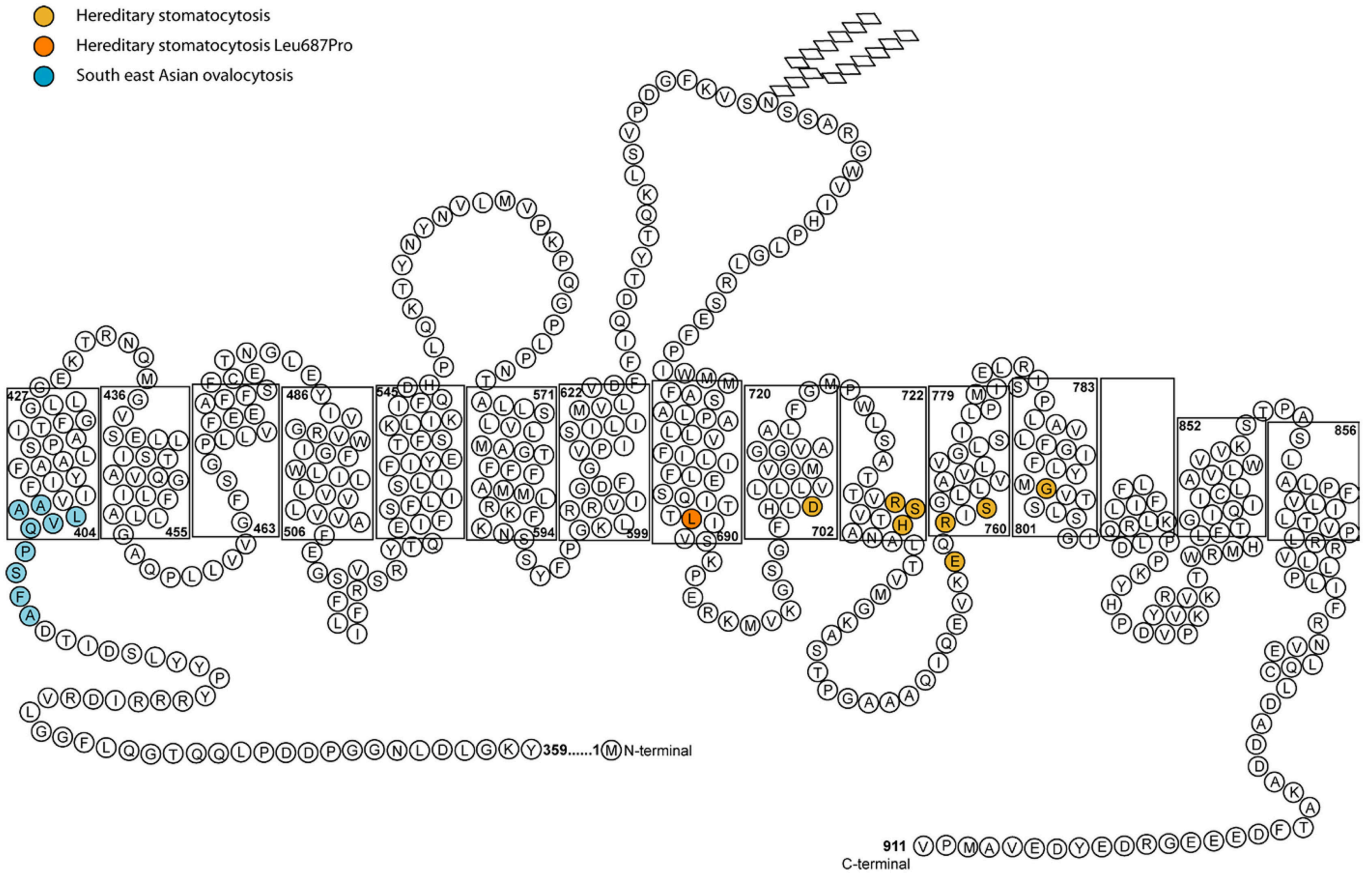
Diagram of the membrane domain of band 3 showing the membrane spans and HSt mutations. The length of the membrane spans mimics that determined from the crystal structure by Arakawa et al. (2015). In the final structure the half transmembrane span 3 and half transmembrane span 10 form a full transmembrane domain (TM) jointly (Arakawa et al., 2015). The nine amino acid residues deleted in SAO band 3 are shown in blue. The amino acid residues associated with HSt are shown in orange. The Asp705Tyr (TM9), Arg730Cys (TM10), Ser731Pro (TM10), His734Arg (TM10) Glu758Lys (TM11), Arg760Gln (TM11), and Ser762Arg (TM11) mutations all occur close to the anion binding site of band 3. The Gly796Arg mutation is in TM12 and may affect helix-helix packing. The Leu687Pro mutation is near the C-terminus of TM8 which is at the interface of the core and gate domains. Movement between these two domains is required for anion transport and substitution of Leu687 with a proline residue may restrict this movement (Arakawa et al., 2015).

All of the band 3 HSt mutations identified so far occur within the transmembrane domain between spans 8 and 12, the region that forms the transport channel of band 3 (Figure 2; Arakawa et al., 2015; Reithmeier et al., 2016). The mutant proteins were shown to be expressed in the CHC patients' RBCs and they induced a cation leak when expressed heterologously in *Xenopus levis* oocytes. The ouabain and bumetanide insensitive cation leak could be inhibited with a range of specific band 3 inhibitors, supporting the hypothesis that the cation leak is mediated directly through the transport channel of band 3 (Bruce et al., 2005). There is variability in the phenotype depending on the mutation. Two mutations were initially identified as corresponding to the classic CHC phenotype: Ser731Pro and His734Gln (Bruce et al., 2005), both occurring very close to each other in transmembrane domain 10 of band 3 (Arakawa et al., 2015). His734 is an essential residue for anion transport and Ser731 lie close to the transport site (Arakawa et al., 2015). Further CHC mutations have since been reported in the same region of the protein, including Ser762Arg (Guizouarn et al., 2011) in span 11 of band 3. This residue interacts with His734 in TM10 (Arakawa et al., 2015; Reithmeier et al., 2016).

One subtype within the cryohydrocytosis group referred to as HS-LTL (hereditary spherocytosis with low temperature leak) results from the mutation Asp705Tyr or Arg760Gln (Bruce et al., 2005) and shows some overlap with the hereditary spherocytosis (HS) group of disorders. In those disorders the vertical link between the RBC cytoskeleton and the membrane is disrupted because of defects in the structurally important proteins band 3, protein 4.2 or ankyrin. In typical HS (without a cation leak) the mutant band 3 may be too misfolded to reach the membrane at all, or the mutant mRNA is unstable and is degraded. The remaining wild type allele produces functional band 3, but not in sufficient quantity to fully compensate for the lost structural interactions. The weakening of the link between the membrane and the underlying cytoskeleton results in the loss of membrane, producing small and dense spherocytic cells. In HS-LTL there is a deficiency in the vertical interactions between the cytoskeleton and band 3 in the membrane, resulting in the same spherocytic morphology, but the difference is that some of the mutant band 3 is successfully trafficked to the membrane, despite being misfolded. Once expressed at the plasma membrane the mutant protein causes a RBC cation leak.

#### Southeast asian ovalocytosis

Southeast Asian Ovalocytosis (SAO) cells are morphologically abnormal, showing large oval cells often with double slits on blood films. The mutation in SAO band 3 is a 9 amino acid deletion at residues 400–408 (Tanner et al., 1991) (Figure 2). This region is situated close to the boundary of the large cytoplasmic N-terminal domain and the transmembrane domain and it is known to affect the normal folding of band 3 (Kuma et al., 2002). Like CHC band 3, the SAO band 3 is expressed in the RBC membrane but is misfolded and cannot transport anions. Early *in vitro* studies of SAO RBCs indicated that these cells were resistant to invasion by the malaria parasite, *Plasmodium falciparum* (Hadley et al., 1983). However, further experimental study showed that a significant part of this effect is caused by depletion in the cellular levels of ATP upon storage of these cells (Dluzewski et al., 1992). This is an effect resulting from a cold-induced cation leak (Bruce et al., 1999), which is almost indistinguishable from that observed in CHC (Guizouarn et al., 2011). However, the SAO mutation has been shown to confer protection from cerebral malaria—a serious complication that can arise from *P. falciparum* infection (Allen et al., 1999), and this could explain the prevalence of SAO in low-lying parts of Papua New Guinea and South East Asia.

#### Other HSt caused by band 3 mutations

HSt Blackburn is another distinct type of cation leak with a characteristic temperature-dependence. This has previously been described as the “shallow slope” type, where cells exhibit a cation leak 4–5 times greater than normal cells at 37°C, but as the temperature lowers only a small decrease in this leak is observed (Figure 1C; Coles and Stewart, 1999). This phenotype is associated with a band 3 point mutation, Leu687Pro, which prevents normal anion exchange function and produces a cation leak when expressed in *X. levis* oocytes (Bruce et al., 2005). Leu687 is at the C-terminus of TM8, which packs against TM3 and TM10 (Figure 2). TM8 is at the interface of the core and gate domains of band 3 (Arakawa et al., 2015) and therefore key to the normal functioning of band 3.

Band 3 New Haven (Glu758Lys) has been reported in 2 patients (Stewart et al., 2010). Expressing this mutant band 3 in oocytes produced anion exchange activity only when GPA was co-expressed, but the cation leak associated with its expression was GPA-independent. These characteristics are in contrast to other HSt band 3 mutations, where anion exchange was completely abolished. The same group also reported a further band 3 mutation in HSt (Arg730Cys) (Stewart et al., 2011). The temperature-dependence of the leaks caused by these two mutations were not characterized.

Band 3 Ceinge (Gly796Arg) is an interesting HSt variant, as this pedigree exhibited signs of dyserythropoiesis and no mutations were found in *CDAN1*, the gene associated with chronic dyserythropoietic anemia (CDA) type 1. Analysis of the membranes also revealed increased tyrosine phosphorylation of band 3 and other membrane proteins (Iolascon et al., 2009).

### RhAG mutations

#### Overhydrated hereditary stomatocytosis

The most severe cation leak in the hereditary stomatocytoses can be found in a condition known as overhydrated hereditary stomatocytosis (OHSt). At 37°C the cation leak can be as high as 40 times greater than normal; the temperature dependence profile shows a simple pattern of increasing leak with increasing temperature (Figure 1A; Coles and Stewart, 1999). Heterozygous mutations in *RHAG* are associated with this disorder—Phe65Ser and Ile61Arg—and again these fall within a transmembrane span (Bruce et al., 2009). This gene encodes the Rh-associated glycoprotein (RhAG), which is distantly related to ammonia transporters in bacteria and yeast (Marini et al., 1997). Molecular modeling of the mutant RhAG suggests that the Phe65Ser mutation causes a significant widening of the central transport channel, converting it into a non-selective cation pore (Bruce et al., 2009). Ile61Arg is also predicted to widen the central channel, but not to the same extent. It was noted that larger structural changes may occur, which would allow passage of cations. In addition, there is experimental evidence that the OHSt mutation Phe65Ser prevents the transport of ammonia, which is thought to be the normal substrate of RhAG (Genetet et al., 2012).

A metabolomics study of four individuals with OHSt has provided an insight into the downstream effects of this RhAG mutation (Darghouth et al., 2011). The authors found that the glycolytic pathway is overactive in OHSt RBCs, with a build-up of glycolysis end products. This is consistent with an increased energy demand in OHSt cells, possibly resulting from elevated activity of the Na,K-ATPase attempting to maintain normal cation gradients. An unexpected finding in the metabolomics study was an alteration in levels of certain members of oxidation pathways, raising the possibility that OHSt RBCs may also suffer from previously unrecognized oxidative stress (Darghouth et al., 2011).

#### Stomatin

Stomatin first attracted attention when it was observed to be deficient in the membranes of overhydrated stomatocytosis RBCs, and was named after the disease (Lande et al., 1982; Stewart et al., 1992). Initially it appeared that the disease must be caused by the absence of this protein, however further investigation revealed that stomatin deficiency was a secondary effect of the disorder (Fricke et al., 2003). It is now known that the primary defect is mutation in RhAG. Since then, a second type of hereditary stomatocytosis has been observed to be deficient in stomatin (sdCHC, see below).

Interestingly, the levels of stomatin are also significantly higher in dog RBCs of the “high K” phenotype and its expression can be used as a marker for the phenotype (Komatsu et al., 2010).

The precise role of stomatin in the RBC has become a long-running debate, although potential roles have been plentiful. Studies using a wide variety of organisms have provided evidence that stomatin is involved in cholesterol binding, actin binding, vesiculation and regulation of ion channels. In species unable to synthesize vitamin C *de novo*, stomatin converts GLUT1 into a transporter of oxidized vitamin C (dehydroascorbic acid; DHA), which is crucial for its recycling (Montel-Hagen et al., 2008).

Cross-linking experiments also implicate stomatin in RBC protein complexes with GLUT1, band 3, aquaporin-1 and other membrane transporters and proteins (Rungaldier et al., 2013). Stomatin is able to form higher order homo-oligomeric complexes in the RBC membrane and probably acts as a scaffolding protein (Salzer and Prohaska, 2001).

#### Rh_null_

The molecular basis of Rh_null_ (regulator type) is mutation in both alleles of *RHAG*, resulting in the absence of RhAG and its closely associated partner proteins from the RBC membrane. Rh_null_ (amorph type) occurs when an RhD-negative individual also has mutations in both alleles of *RHCE*, in which case RhAG is only weakly expressed (Cartron, 1999). This deficiency of the Rh protein complex is associated with a number of as yet unexplained features including altered membrane phospholipid asymmetry, occasional stomato-spherocytic morphology and a mild cation leak (reviewed by Cartron, 1999). Patients usually experience chronic mild to moderate hemolytic anemia.

Analysis of morphologically normal Rh_null_ RBCs revealed normal cation and water content but an increase in the basal membrane permeability to potassium and the ouabain and bumetanide sensitive potassium permeability. Consistent with the observed increase in active potassium transport, the number of Na,K-ATPase molecules in the Rh_null_ RBCs was greater than normal by 35–45% (Lauf and Joiner, 1976). The temperature-dependence of the leak has not been established.

There is evidence that protein-lipid interactions are altered in Rh_null_ RBCs (Dorn-Zachertz and Zimmer, 1981). Band 3 has been shown to interact strongly with RhAG, so its absence is likely to disrupt the correct formation of the band 3 macrocomplex (Bruce et al., 2003). More work is needed to elucidate the mechanism behind the increased cation permeability previously observed in 2 cases of Rh_null_. However, the lack of availability of this extremely rare blood type is restrictive for comprehensive studies.

### GLUT1 mutations

Stomatin-deficient cryohydrocytosis (sdCHC) is a very rare type of HSt, and has only been reported in 3 patients to date (Bawazir et al., 2012). Alongside a RBC cation leak that is 10 times greater than normal at 37°C (Figure 1B) these patients also suffer from numerous non-hematological symptoms that do not occur in any of the other types of hereditary stomatocytosis. These include cataracts and a debilitating neurological phenotype comprising seizures and severe learning difficulties. The gene responsible has been identified as *SLC2A1*, encoding glucose transporter isoform 1 (GLUT1). GLUT1 is the major glucose transporter expressed in RBCs and at the blood-brain barrier. Two distinct mutations in *SLC2A1* have been found in sdCHC patients (Flatt et al., 2011). In the Montpellier pedigree a single amino acid substitution mutation was found (Gly286Asp), whilst the San Francisco pedigree exhibited a single amino acid deletion (Ile435 or Ile436). In an analogous situation to the RhAG mutants in OHSt and some of the band 3 mutants in CHC, the GLUT1 mutants lost their ability to transport their usual substrate, and instead induced a cation leak when expressed heterologously in *X. levis* oocytes.

GLUT1 mutations have also been shown to result in a RBC cation leak in one pedigree suffering from another condition called paroxysmal exertion-induced dyskinesia (PED) (Weber et al., 2008). In PED involuntary movements are brought on by exercise, and it is not normally associated with a RBC cation leak. In this case the mutation in GLUT1 was identified as a deletion of residues 282–285, which is immediately adjacent to the substitution mutation identified in sdCHC. This mutation occurs within transmembrane span 7, which has been shown to be crucial for transport function. Modeling of the mutant protein suggested that the central pore is widened. Unlike in sdCHC, these RBCs exhibited spiky protrusions (echinocytosis), which are often associated with cellular dehydration. The permeability of the RBCs to sodium, potassium and calcium was increased, raising the possibility that calcium influx might be sufficient to activate the Gardos channel and trigger rapid potassium efflux and cellular dehydration, explaining the echinocytosis (Weber et al., 2008).

Mutations in GLUT1 are also associated with GLUT1-deficiency syndrome (GLUT1-DS). This disorder shows some heterogeneity in its severity and symptoms (Brockmann, 2009). However, there does appear to be a correlation between the effect on glucose transport and clinical severity (Rotstein et al., 2010). Inactivating mutations in one allele result in ~50% reduction in glucose transport, and these are the most common form of GLUT1-DS. Other missense mutations appear to impact glucose transport in a minor way, resulting in an asymptomatic or mild phenotype. Compound heterozygosity or homozygosity for a “mild” mutation could therefore result in a severe phenotype where glucose transport is reduced by more than 50%. A reduction of 75% or more is likely to be embryonic lethal (Rotstein et al., 2010).

### ABCB6 mutations

The condition, familial pseudohyperkalemia (FP), is a very mild form of HSt that can often be asymptomatic. The condition was first reported in 1979 (Stewart et al., 1979), although FP in this cohort was later shown to map to the 16q23-ter locus (Iolascon et al., 1999) and result from a mutation in PIEZO1 (Andolfo et al., 2013b). A detailed analysis of the temperature sensitivity of the potassium flux was described in 1985 (Meenaghan et al., 1985). At the physiological temperature of 37°C the cation leak is practically indistinguishable from normal, being only 1.1–1.5 times the normal rate (Stewart, 2004). The close to normal cation permeability at 37°C means that samples of FP blood when tested immediately after venesection show normal hematological parameters. However, after cooling to room temperature and/or refrigeration the cation leak is “activated” and a rise in plasma potassium occurs after a period of cool or room temperature storage. Although all FP cases share this feature, the heterogeneity in the temperature-dependence of their cation leaks means that the degree of potassium efflux varies between the different subtypes at different temperatures.

The temperature dependences of the cation leaks in the FP pedigrees are shown in Figure 1, and include U-shaped (Figure 1B), shallow slope (Figure 1C) and shoulder types (Figure 1D). All show a cation leak very similar to the control at 37°C which increases as the temperature decreases. The molecular bases of a number of FP pedigrees have been determined recently, with mutations in the ATP-binding cassette family member B6 (ABCB6) identified as responsible (Andolfo et al., 2013a, 2016; Bawazir et al., 2014). The ABCB6 protein is a porphyrin transporter that is considered to be a mitochondrial protein, but has also been detected in lysosomes and the RBC plasma membrane (Kiss et al., 2012). Indeed, it has also been discovered that ABCB6 carries the antigens of the Langereis blood group system (Helias et al., 2012).

Two of the *ABCB6* mutations affect the same residue in the protein (R375Q and R375W) and both of these changes result in the same temperature-dependence profile in their cation leak (shoulder type with a peak at 10°C, (Figure 1D). A different mutation in ABCB6 is associated with the Cardiff pedigree (R723Q), whose cation leak shows a U-shaped temperature-dependence (Figure 1B). Hence, the cation leak in this type of FP only becomes apparent at temperatures lower than 20°C and further increases as the temperature decreases. Although the vast majority of FP mutations seem to be very rare, the SNP database suggests that the R723Q mutation has a frequency of 1 in 500 (Bawazir et al., 2014). The temperature-dependent activation of these leaks during the processing and storage of RBC concentrates for use in transfusion medicine should be a concern when selecting units for transfusion of high-risk patients such as neonates (Bawazir et al., 2014).

Three further mutations in ABCB6 have been reported in FP families. The Arg276Trp mutation has been found alone, and in trans to the Arg723Gln mutation, in two separate families (Andolfo et al., 2016), and the Val454Ala mutation has been found in a Bolivian family (Andolfo et al., 2016). Andolfo et al. present data to show that the Arg276Trp and Val454Ala mutations increase cation permeability of HEK293 cells over-expressing these mutant ABCB6 proteins, and show evidence of a slight increase in cation permeability in FP RBCs with the Arg276Trp mutation. Interestingly the family with the Arg276Trp mutation is the Irish family from Omagh that were initially classified as DHSt with FP (Table 1; Stewart et al., 1996). The temperature profile was assigned as parallel (Figure 1E) but may fit better in the U-shaped classification (Figure 1B) as the temperature profile is similar to the FP-Cardiff profile although milder (note the different Y-axis scales on Figures 1B,D). It would not be expected that the Arg276Trp mutation would cause a large difference in cation permeability because it is classified as “benign” in the Single Nucleotide Polymorphism database: https://www.ncbi.nlm.nih.gov/projects/SNP/snp_ref.cgi?rs=57467915 and this mutant has a fairly high population frequency in the European population. This would therefore be expected to have caused more problems with blood storage and transfusion than have been reported. The high population frequency of this mutation underlines the fact that FP is an under-diagnosed condition (Andolfo et al., 2018a,b).

### Unresolved—HST woking

An unusual case of hereditary stomatocytosis has been reported in which there is a significant cation leak (5 times greater than normal, but with a normal temperature dependence pattern), yet there was minimal hemolysis observed and nearly normal osmotic fragility (Jarvis et al., 2001). The affected offspring of the propositus experienced an even milder phenotype. The authors speculated that the discrepancy between the grossly abnormal cation content and mild phenotype was because the sodium and potassium abnormalities were relatively balanced (Jarvis et al., 2001). The genetic basis for this variant is unknown and has not been mapped.

## HST mutations in affecting the regulation of cation channels

### PIEZO1 mutations

Dehydrated hereditary stomatocytosis (DHSt; also known as hereditary xerocytosis) is a condition, similar to FP that falls at the milder end of the cation leaky spectrum. DHSt RBCs exhibit an elevation in their passive cation leak at 37°C of around twice normal, and the temperature-dependence profiles of the leak (shown in Figure 1, Table 1) have been reported as shallow slope (Figure 1C), parallel (Figure 1D), sigmoidal (Figure 1E) or flat to 30°C (Figure 1G). This can mean that the relative increase in cation permeability of DHSt cells is augmented at lower temperatures. Indeed, all four panels show an increased cation leak relative to the control at 20°C, most markedly in the parallel and flat to 30°C profiles, however only the parallel profile shows an increased cation leak at 4°C (Figure 1). It should be noted that most of the profiles shown in the shallow slope graph (Figure 1C) are the HSt Blackburn type caused by mutations in *SLC4A1*. With hind sight, it may be that the Edinburgh profile would fit better as a mild form of the parallel profile. This tendency for DHSt RBCs to have a very minor difference in cation permeability at 37°C but maintain an increased cation leak, relative to the control, at lower temperature explains why DHSt is so often associated with pseudohyperkalemia.

The nature of the cation leak in DHSt cells is imbalanced, in that there is a greater loss of potassium from the cells than the increase in sodium, which leads to dehydration of the RBC. The dehydrated cells are accordingly more osmotically resistant than controls, and also have an unexplained increased susceptibility to oxidants (Harm et al., 1979). Individuals with DHSt exhibit mild hemolytic anemia with increased reticulocytes and macrocytosis. Unlike in other hemolytic anemias, splenectomy is not recommended for patients with DHSt because it often results in thrombo-embolic events (Stewart et al., 1996).

After a long search for the molecular basis of DHSt a breakthrough was finally made, where mutations in the gene encoding PIEZO1, *FAM38A*, were discovered to be associated with the disorder (Zarychanski et al., 2012). The PIEZO1 protein forms part of a mechanosensitive transduction channel (Coste et al., 2010). PIEZO1 is a very large protein, predicted to cross the membrane 36–39 times and has been identified in RBC membranes using mass spectrometry and Western blotting methods (Zarychanski et al., 2012; Andolfo et al., 2013b). It has been shown to induce cationic fluxes in response to mechanical stimulation in eukaryotic cells (Coste et al., 2010). Expression of wild type PIEZO1 in *X. levis* oocytes resulted in mechano-stimulated currents that were not observed in uninjected oocytes. These currents were also observed in PIEZO1-expressing oocytes subjected to hypotonic and hypertonic induced swelling and shrinkage, respectively (Andolfo et al., 2013b). Comparison of the conductances of normal and DHSt (with mutation R2456H) RBCs using cell-attached recording showed that DHSt cells exhibited a conductance that could be inhibited with tarantula toxin, GsMTx-4, supporting the hypothesis that the cation leak is mediated by PIEZO1 (Andolfo et al., 2013b). Expression of DHSt mutant PIEZO1 proteins in HEK293T cells revealed that all six of the tested mutations result in slower inactivation of the current (Albuisson et al., 2013). This gain-of-function pattern would be consistent with the observed dehydrated phenotype. Conversely knockdown of PIEZO1 in zebrafish or mouse results in swollen cells (Faucherre et al., 2014; Cahalan et al., 2015).

It therefore seems probable that different mutations in the two genes known to be associated with DHSt and FP can result in highly similar phenotypes. Equally, different mutations within the same gene can result in distinct phenotypes, as has also been seen in the other stomatocytosis variants. For example, some mutations affect the transport ability of the proteins while others do not, and the two *RHAG* mutations resulting in OHSt give rise to subtly different phenotypes (Table 1). Although the ability of PIEZO1 or ABCB6 to transport their usual substrates has not been fully characterized in DHSt and FP RBCs, it is possible that inhibited transport may affect the resulting phenotype. This is particularly interesting in the case of PIEZO1, which has been shown to act as a stretch sensor in RBCs and to play a key role in RBC volume regulation (Zarychanski et al., 2012). Recent studies have shown that the DHSt mutations in PIEZO1 cause a partial gain-of-function phenotype (Albuisson et al., 2013; Andolfo et al., 2013b). Generation of mechanically activated currents inactivate more slowly than wild type. However in addition to delayed channel inactivation, additional alterations have been found in mutant PIEZO1 channel kinetics, differences in response to osmotic stress, and altered membrane protein trafficking, predicting variant alleles that worsen or ameliorate erythrocyte hydration (Glogowska et al., 2017).

DHSt is occasionally associated with perinatal ascites (buildup of fluid in the baby's abdominal cavity) and/or nuchal translucency (buildup of fluid at the back of the baby's head observed during ultrasound scans) (Entezami et al., 1996; Ami et al., 2009). Andolfo et al. (2013b) investigated the tissue expression pattern of PIEZO1 and found that it was expressed in the peritoneal lymphatic vessels in fetal, but not adult, samples. This expression pattern is consistent with the observed phenotype of edema that spontaneously self-corrects just after birth (Andolfo et al., 2013b). It is of interest that the mutations detected in DHSt with associated ascites have also been found in DHSt cases where ascites has not been reported. It is possible that this is an under-reported condition, and that some cases have not been detected or diagnosed, especially when the edema resolves prenatally (personal communication from Prof Jean Delaunay, 2013). The severity of the edema appears to vary between cases, even those with the same mutation in PIEZO1, and may suggest the presence of a modifying gene. In recent years, numerous PIEZO1 mutations have been reported associated with DHSt alone, DHSt with pseudohyperkalemia and /or with edema (see Table 1).

### Gardos channel mutations

After the breakthrough discovery that mutations in PIEZO1 cause various forms of DHSt, several groups have recently published studies showing that mutations in the Gardos channel (heterozygous substitutions Val282Glu, Val282Met, Arg352His), encoded by the gene *KCNN4*, are associated with DHSt (Andolfo et al., 2015; Glogowska et al., 2015; Rapetti-Mauss et al., 2015). Functional analysis of the R352H mutant suggested that it was 10-fold more sensitive to activation by calcium and also remained in the open conformation longer than the wild type protein (Rapetti-Mauss et al., 2015).

These findings have led to the hypothesis that DHSt result from a malfunction in the regulation of the joint activity of the Gardos channel and PIEZO1. In wild-type RBC, passage through constricted splenic slits or small capillary beds is associated with transient calcium-mediated adaptions (Danielczok et al., 2017). It is proposed that this transient increase in intracellular calcium is mediated by the opening of the PIEZO1 channel, which opens in response to the shear stress of the constricted space. The rise in intracellular calcium then causes the Gardos channel to open with the loss of potassium, followed by chloride ions and water, thus dehydrating the cell. This would make the Gardos channel the essential determinant of RBC dehydration in HSt (Rapetti-Mauss et al., 2017), regardless of whether the mutation was in PIEZO1 causing a prolonged influx of calcium or in KCNN4 making the Gardos channel more sensitive to intracellular calcium.

Despite the discovery of the genes responsible for DHSt, it is still not clear why many individuals with DHSt have been reported to exhibit a propensity to iron overload (Syfuss et al., 2006). It has been suggested that dyserythropoiesis in these conditions may play a role but this is yet to be confirmed (Andolfo et al., 2015).

## Origin of the cation leak in the hereditary stomatocytoses

Once it was discovered that band 3 mutations result in a cation leak, the mechanism of the leak was debated. In the initial report it was suggested that cation movements were conducted directly though the mutant band 3, and further investigation of the band 3 CHC mutations supported this proposal (Bruce et al., 2005; Guizouarn et al., 2007). However, Bogdanova et al. reported increased cation transport via the K(Na)/H exchanger and the K,Cl cotransporter in CHC RBCs leading to the proposal of an alternative mechanism whereby the mutant band 3 activates other endogenous transporters in the membrane, causing the cation imbalance (Bogdanova et al., 2010; Stewart et al., 2010). It is interesting to note that cation-leaky band 3 point mutations are mainly clustered around the same region in the protein, between transmembrane span 8 and span 12, the region of the protein intimately involved in anion transport (Arakawa et al., 2015) (Figure 2). The exception to this is the Leu687Pro mutation which is in TM 8 at the interface of the core and gate domains. This mutation may obstruct the movement between these two domains and thereby affect anion exchange and the correct packing of the protein (Figure 2).

Modeling RhAG based on the structure of *Nitrosomonas europea* Rh50 protein predicted that the OHSt mutations resulted in widening of the central channel and its conversion into a cation pore (Bruce et al., 2009). The leak in OHSt increases with temperature in a linear pattern, consistent with the hypothesis of the conversion of RhAG into a constitutively open cation channel. The more complex temperature-dependence of the other HSt leaks might suggest a less clear cut mechanism of cation leak. In the cryohydrocytosis types, the leak is even enhanced at lower temperatures. The GLUT1 mutations responsible for sdCHC have been modeled, with Gly286Asp predicted to result in a more rigid protein structure and Ile435DEL predicted to result in longer range conformational changes that are hard to model accurately (Flatt et al., 2011). This distinct temperature dependence and lack of structural evidence for the formation of a pore suggest that the cation leak may not be conducted directly through the transport channel of GLUT1. Instead, the leak may be mediated via altered protein-protein or protein-lipid interactions. Both band 3 and GLUT1 have been shown to participate in larger protein complexes (Bruce et al., 2003; Khan et al., 2008), so it is conceivable that small alterations in their structures, and resultant macro-structures, could provide gaps through which the leak could occur. Alternatively, small alterations in protein-lipid interactions could provide a pathway for cations across the membrane. It is becoming more evident that the surfaces of proteins contain specific lipid binding sites that are important for the proper expression and function of the protein in the membrane (reviewed by Palsdottir and Hunte, 2004). Perturbation of these interactions could be thought of as a weakening of the lipid “seal” around the protein. At lower temperatures membranes become more rigid, which could exacerbate the poor fit of lipids around mutant proteins and their multi-protein complexes and provide an explanation for how and why the leaks worsen at low temperatures.

Given that mutations in 4 different large multi-spanning membrane proteins have now been associated with these cation leaky conditions it seems unlikely that a distinct cation transporter pathway is being activated and causing the cation imbalance. Rather, it appears to be the general rule that mutation in any large multi-spanning membrane protein can provoke a cation leak if it is expressed at the RBC plasma membrane.

More recently the discovery and characterization of mutations in PIEZO1 and the Gardos channel in DHSt have been made. This has revealed a second, alternative mechanism behind HSt, in which an over-active mutant cation channel is responsible for the observed increase in membrane permeability to cations.

## Other incidences of stomatocytic morphology

The presence of stomatocytes in the blood is not exclusive to the cation-leaky disorders. Phytosterolemia is a recessively-inherited condition in which cholesterols and plant-derived sterols are absorbed from the gut in a deregulated manner. The excess of phytosterols in the blood can result in stomatocytosis and large platelets (Rees et al., 2005). It is thought that the phytosterols are able to partition into the membranes of circulating RBCs and selectively expand the inner leaflet of the bilayer, inducing an inward curve in the membrane that leads to the adoption of stomatocytic morphology. Indeed, the quantity of stomatocytic cells can vary widely, and they are often not overtly predominant in blood smears from hereditary stomatocytosis pedigrees. Blood smears commonly appear normal in familial pseudohyperkalemia and in DHSt abnormal cells are principally target cells, not stomatocytes.

## Summary

At present the evidence strongly suggests that there are two distinct mechanisms underlying the pathological increase in cation permeability of the RBC membrane in hereditary stomatocytosis. Modifications in a variety of multi-spanning membrane proteins including band 3, RhAG, GLUT1 and ABCB6 can result in formation of a cation pore or otherwise disrupt the membrane to allow unregulated cation movement across the membrane. Alternatively, modifications to existing cation channels such as PIEZO1 and the Gardos channel can alter their activation and deactivation kinetics, leading to increased opening and allowing greater cation fluxes than in wild type. The degree and character of the “leakiness” in these conditions can vary greatly, illustrated by the diverse phenotypes found in HSt.

## Author contributions

Both authors conceived and developed the ideas in this review. JF wrote the paper and LB edited and updated it.

### Conflict of interest statement

The authors declare that the research was conducted in the absence of any commercial or financial relationships that could be construed as a potential conflict of interest.

## References

[B1] Albuisson J. Murthy S. E. Bandell M. Coste B. Louis-Dit-Picard H. Mathur J.. (2013). Dehydrated hereditary stomatocytosis linked to gain-of-function mutations in mechanically activated PIEZO1 ion channels. Nat. Commun. 4:1884. 10.1038/ncomms2899 23695678 PMC3674779

[B2] Allen S. J. O'Donnell A. Alexander N. D. Mgone C. S. Peto T. E. Clegg J. B.. (1999). Prevention of cerebral malaria in children in Papua New Guinea by southeast Asian ovalocytosis band 3. Am. J. Trop. Med. Hyg. 60, 1056–1060. 10.4269/ajtmh.1999.60.1056 10403343

[B3] Ami O. Picone O. Garçon L. Castel C. Guitton C. Delaunay J.. (2009). First-trimester nuchal abnormalities secondary to dehydrated hereditary stomatocytosis. Prenat. Diagn. 29, 1071–1074. 10.1002/pd.2342 19655317

[B4] Andolfo I. Alper S. L. De Franceschi L. Auriemma C. Russo R. De Falco L.. (2013b). Multiple clinical forms of dehydrated hereditary stomatocytosis arise from mutations in PIEZO1. Blood 121, 3925–3935. 10.1182/blood-2013-02-482489 23479567

[B5] Andolfo I. Alper S. L. Delaunay J. Auriemma C. Russo R. Asci R.. (2013a). Missense mutations in the ABCB6 transporter cause dominant familial pseudohyperkalemia. Am. J. Hematol. 88, 66–72. 10.1002/ajh.23357 23180570

[B6] Andolfo I. Manna F. De Rosa G. Rosato B. E. Gambale A. Tomaiuolo G.. (2018a). PIEZO1-R1864H rare variant accounts for a genetic phenotype-modifier role in dehydrated hereditary stomatocytosis. Haematologica 103, e94–e97. 10.3324/haematol.2017.180687 29191841 PMC5830381

[B7] Andolfo I. Russo R. Gambale A. Iolascon A. (2018b). Hereditary stomatocytosis: an underdiagnosed condition. Am. J. Hematol. 93, 107–121. 10.1002/ajh.24929 28971506

[B8] Andolfo I. Russo R. Manna F. De Rosa G. Gambale A. Zouwail S.. (2016). Functional characterization of novel ABCB6 mutations and their clinical implications in familial pseudohyperkalemia. Haematologica 101, 909–917. 10.3324/haematol.2016.142372 27151991 PMC4967569

[B9] Andolfo I. Russo R. Manna F. Shmukler B. E. Gambale A. Vitiello G.. (2015). Novel Gardos channel mutations linked to dehydrated hereditary stomatocytosis (Xerocytosis). Am. J. Hematol. 90, 921–926. 10.1002/ajh.24117 26178367

[B10] Arakawa T. Kobayashi-Yurugi T. Alguel Y. Iwanari H. Hatae H. Iwata M.. (2015). Crystal structure of the anion exchanger domain of human erythrocyte band 3. Science 350, 680–684. 10.1126/science.aaa4335 26542571

[B11] Archer N. M. Shmukler B. E. Andolfo I. Vandorpe D. H. Gnanasambandam R. Higgins J. M.. (2014). Hereditary xerocytosis revisited. Am. J. Hematol. 89, 1142–1146. 10.1002/ajh.23799 25044010 PMC4237618

[B12] Bae C. Gnanasambandam R. Nicolai C. Sachs F. Gottlieb P. A. (2013). Xerocytosis is caused by mutations that alter the kinetics of the mechanosensitive channel PIEZO1. Proc. Natl. Acad. Sci. U.S.A. 110, E1162–E1168. 10.1073/pnas.1219777110 23487776 PMC3606986

[B13] Bae C. Sachs F. Gottlieb P. A. (2011). The mechanosensitive ion channel Piezo1 is inhibited by the peptide GsMTx4. Biochemistry 50, 6295–6300. 10.1021/bi200770q 21696149 PMC3169095

[B14] Basu A. P. Carey P. Cynober T. Chetty M. Delaunay J. Stewart G. W.. (2003). Dehydrated hereditary stomatocytosis with transient perinatal ascites. Arch. Dis. Child. Fetal Neonatal Ed. 88, F438–F439. 10.1136/fn.88.5.F438 12937055 PMC1721605

[B15] Bawazir W. M. Flatt J. F. Wallis J. P. Rendon A. Cardigan R. A. New H. V.. (2014). Familial pseudohyperkalemia in blood donors: a novel mutation with implications for transfusion practice. Transfusion 54, 3043–3050. 10.1111/trf.12757 24947683

[B16] Bawazir W. M. Gevers E. F. Flatt J. F. Ang A. L. Jacobs B. Oren C.. (2012). An infant with pseudohyperkalemia, hemolysis, and seizures: cation-leaky GLUT1-deficiency syndrome due to a SLC2A1 mutation. J. Clin. Endocrinol. Metab. 97, E987–E993. 10.1210/jc.2012-1399 22492876

[B17] Beneteau C. Thierry G. Blesson S. Le Vaillant C. Picard V. Béné M.. (2014). Recurrent mutation in the PIEZO1 gene in two families of hereditary xerocytosis with fetal hydrops. Clin. Genet. 85, 293–295. 10.1111/cge.12147 23581886

[B18] Bennekou P. Christopherson P. (2003). Ion channels in Red Cell Membrane Transport, eds Bernhardt I. Ellory J. C. (Berlin; Heidelberg: Springer-Verlag), 139–152.

[B19] Bernhardt I. Weiss E. (2003). Passive membrane permeability for ions and the membrane potential in Red Cell Membrane Transport, eds Bernhardt I. Ellory J. C. (Berlin; Heidelberg: Springer-Verlag), 83–109.

[B20] Bogdanova A. Goede J. S. Weiss E. Bogdanov N. Bennekou P. Bernhardt I.. (2010). Cryohydrocytosis: increased activity of cation carriers in red cells from a patient with a band 3 mutation. Haematologica 95, 189–198. 10.3324/haematol.2009.010215 20015879 PMC2817020

[B21] Brockmann K. (2009). The expanding phenotype of GLUT1-deficiency syndrome. Brain Dev. 31, 545–552. 10.1016/j.braindev.2009.02.008 19304421

[B22] Bruce L. J. Beckmann R. Ribeiro M. L. Peters L. L. Chasis J. A. Delaunay J.. (2003). A band 3-based macrocomplex of integral and peripheral proteins in the RBC membrane. Blood 101, 4180–4188. 10.1182/blood-2002-09-2824 12531814

[B23] Bruce L. J. Guizouarn H. Burton N. M. Gabillat N. Poole J. Flatt J. F.. (2009). The monovalent cation leak in overhydrated stomatocytic red blood cells results from amino acid substitutions in the Rh-associated glycoprotein. Blood 113, 1350–1357. 10.1182/blood-2008-07-171140 18931342

[B24] Bruce L. J. Ring S. M. Ridgwell K. Reardon D. M. Seymour C. A. Van Dort H. M.. (1999). South-east asian ovalocytic (SAO) erythrocytes have a cold sensitive cation leak: implications for *in vitro* studies on stored SAO red cells. Biochim. Biophys. Acta 1416, 258–270. 10.1016/S0005-2736(98)00231-4 9889381

[B25] Bruce L. J. Robinson H. C. Guizouarn H. Borgese F. Harrison P. King M. J.. (2005). Monovalent cation leaks in human red cells caused by single amino-acid substitutions in the transport domain of the band 3 chloride-bicarbonate exchanger, AE1. Nat. Genet. 37, 1258–1263. 10.1038/ng1656 16227998

[B26] Burton N. M. Anstee D. J. (2008). Structure, function and significance of Rh proteins in red cells. Curr. Opin. Hematol. 15, 625–630. 10.1097/MOH.0b013e328311f422 18832935

[B27] Cahalan S. M. Lukacs V. Ranade S. S. Chien S. Bandell M. Patapoutian A. (2015). Piezo1 links mechanical forces to red blood cell volume. Elife. 4. 10.7554/eLife.07370 26001274 PMC4456639

[B28] Carella M. Stewart G. Ajetunmobi J. F. Perrotta S. Grootenboer S. Tchernia G.. (1998). Genomewide search for dehydrated hereditary stomatocytosis (hereditary xerocytosis): mapping of locus to chromosome 16 (16q23-qter). Am. J. Hum. Genet. 63, 810–816. 10.1086/302024 9718354 PMC1377412

[B29] Carli P. Graffin B. Gisserot O. Landais C. De Jaureguiberry J. P. (2007). Recurrence of thromboembolic disease after splenectomy for hereditary xerocytosis. Rev. Med. Interne. 28, 879–881. 10.1016/j.revmed.2007.05.012 17590481

[B30] Cartron J. P. (1999). RH blood group system and molecular basis of Rh-deficiency. Baillieres. Best Pract. Res. Clin. Haematol. 12, 655–689. 10.1053/beha.1999.0047 10895258

[B31] Chan P. C. Calabrese V. Theil L. S. (1964). Species differences in the effect of sodium and potassium ions on the ATPase of erythrocyte membranes. Biochim. Biophys. Acta 79, 424–426. 10.1016/0926-6577(64)90028-2 14163532

[B32] Coles S. E. Chetty M. C. Ho M. M. Nicolaou A. Kearney J. W. Wright S. D.. (1999a). Two British families with variants of the “cryohydrocytosis” form of hereditary stomatocytosis. Br. J. Haematol. 105, 1055–1065. 10554820 10.1046/j.1365-2141.1999.01444.x

[B33] Coles S. E. Ho M. M. Chetty M. C. Nicolaou A. Stewart G. W. (1999b). A variant of hereditary stomatocytosis with marked pseudohyperkalaemia. Br. J. Haematol. 104, 275–283. 10050708 10.1046/j.1365-2141.1999.01191.x

[B34] Coles S. E. Stewart G. W. (1999). Temperature effects on cation transport in hereditary stomatocytosis and allied disorders. Int. J. Exp. Pathol. 80, 251–258. 10.1046/j.1365-2613.1999.00120.x 10607015 PMC2517829

[B35] Coste B. Mathur J. Schmidt M. Earley T. J. Ranade S. Petrus M. J.. (2010). Piezo1 and Piezo2 are essential components of distinct mechanically activated cation channels. Science 330, 55–60. 10.1126/science.1193270 20813920 PMC3062430

[B36] Dagher G. Vantyghem M. C. Doise B. Lallau G. Racadot A. Lefebvre J. (1989). Altered erythrocyte cation permeability in familial pseudohyperkalaemia. Clin. Sci. 77, 213–216. 10.1042/cs0770213 2766660

[B37] Danielczok J. G. Terriac E. Hertz L. Petkova-Kirova P. Lautenschläger F. Laschke M. W.. (2017). Red blood cell passage of small capillaries is associated with transient Ca2^+^-mediated adaptations. Front. Physiol. 8:979. 10.3389/fphys.2017.00979 29259557 PMC5723316

[B38] Darghouth D. Koehl B. Heilier J. F. Madalinski G. Bovee P. Bosman G.. (2011). Alterations of red blood cell metabolome in overhydrated hereditary stomatocytosis. Haematologica 96, 1861–1865. 10.3324/haematol.2011.045179 21859730 PMC3232270

[B39] Dluzewski A. R. Nash G. B. Wilson R. J. Reardon D. M. Gratzer W. B. (1992). Invasion of hereditary ovalocytes by *Plasmodium falciparum in vitro* and its relation to intracellular ATP concentration. Mol. Biochem. Parasitol. 55, 1–7. 10.1016/0166-6851(92)90121-Y 1435863

[B40] Dorn-Zachertz D. Zimmer G. (1981). Different protein-lipid interaction in human red blood cell membrane of Rh positive and Rh negative blood compared with Rhnull. Z Naturforsch. C 36, 988–996. 6275621 10.1515/znc-1981-11-1215

[B41] Endeward V. Cartron J. P. Ripoche P. Gros G. (2006). Red cell membrane CO2 permeability in normal human blood and in blood deficient in various blood groups, and effect of DIDS. Transfus. Clin. Biol. 13, 123–127. 10.1016/j.tracli.2006.02.007 16563834

[B42] Entezami M. Becker R. Menssen H. D. Marcinkowski M. Versmold H. T. (1996). Xerocytosis with concomitant intrauterine ascites: first description and therapeutic approach. Blood 87, 5392–5393. 8652859

[B43] Faucherre A. Kissa K. Nargeot J. Mangoni M. Jopling C. (2014). Piezo1 plays a role in erythrocyte volume homeostasis. Haematologica 99, 70–75. 10.3324/haematol.2013.086090 23872304 PMC4007942

[B44] Fermo E. Bogdanova A. Petkova-Kirova P. Zaninoni A. Marcello A. P. Makhro A. (2017). “Gardos Channelopathy:” a variant of hereditary Stomatocytosis with complex molecular regulation. Sci. Rep. 7:1744 10.1038/s41598-017-01591-w 28496185 PMC5431847

[B45] Flatman P. W. (2002). Regulation of Na-K-2Cl cotransport by phosphorylation and protein-protein interactions. Biochim. Biophys. Acta 1566, 140–151. 10.1016/S0005-2736(02)00586-2 12421545

[B46] Flatt J. F. Guizouarn H. Burton N. M. Borgese F. Tomlinson R. J. Forsyth R. J.. (2011). Stomatin-deficient cryohydrocytosis results from mutations in SLC2A1: a novel form of GLUT1 deficiency syndrome. Blood 118, 5267–5277. 10.1182/blood-2010-12-326645 21791420

[B47] Fricke B. Argent A. C. Chetty M. C. Pizzey A. R. Turner E. J. Ho M. M.. (2003). The “stomatin” gene and protein in overhydrated hereditary stomatocytosis. Blood. 102, 2268–2277. 10.1182/blood-2002-06-1705 12750157

[B48] Fricke B. Jarvis H. G. Reid C. D. Aguilar-Martinez P. Robert A. Quittet P.. (2004). Four new cases of stomatin-deficient hereditary stomatocytosis syndrome: association of the stomatin-deficient cryohydrocytosis variant with neurological dysfunction. Br. J. Haematol. 125, 796–803. 10.1111/j.1365-2141.2004.04965.x 15180870

[B49] Gallagher P. G. (2017). Disorders of erythrocyte hydration. Blood 130, 2699–2708. 10.1182/blood-2017-04-590810 29051181 PMC5746162

[B50] Genetet S. Ripoche P. Picot J. Bigot S. Delaunay J. Armari-Alla C.. (2012). Human RhAG ammonia channel is impaired by the Phe65Ser mutation in overhydrated stomatocytic red cells. Am. J. Physiol. Cell Physiol. 302, C419–C428. 10.1152/ajpcell.00092.2011 22012326

[B51] Gibson J. S. (2003). Comparative physiology of red cell membrane transport, in Red Cell Membrane Transport, eds Bernhardt I. Ellory J. C. (Berlin; Heidelberg: Springer-Verlag), 721–734.

[B52] Glader B. E. Fortier N. Albala M. M. Nathan D. G. (1974). Congenital hemolytic anemia associated with dehydrated erythrocytes and increased potassium loss. N. Engl. J. Med. 291, 491–496. 10.1056/NEJM197409052911003 4851153

[B53] Glogowska E. Lezon-Geyda K. Maksimova Y. Schulz V. P. Gallagher P. G. (2015). Mutations in the Gardos channel (KCNN4) are associated with hereditary xerocytosis. Blood 126, 1281–1284. 10.1182/blood-2015-07-657957 26198474 PMC4566808

[B54] Glogowska E. Schneider E. R. Maksimova Y. Schulz V. P. Lezon-Geyda K. Wu J.. (2017). Novel mechanisms of PIEZO1 dysfunction in hereditary xerocytosis. Blood 130, 1845–1856. 10.1182/blood-2017-05-786004 28716860 PMC5649553

[B55] Gore D. M. Chetty M. C. Coles S. C. Gover P. M. Stewart G. W. (2002a). Hereditary spherocytosis with a low temperature Na/K leak and thrombosis. Br. J. Haematol. 117(Suppl. 1), 9–10.

[B56] Gore D. M. Chetty M. C. Fisher J. Nicolaou A. Stewart G. W. (2002b). Familial pseudohyperkalaemia Cardiff: a mild version of cryohydrocytosis. Br. J. Haematol. 117, 212–214. 11918557 10.1046/j.1365-2141.2002.03376.x

[B57] Gore D. M. Layton M. Sinha A. K. Williamson P. J. Vaidya B. Connolly V.. (2004). Four pedigrees of the cation-leaky hereditary stomatocytosis class presenting with pseudohyperkalaemia. Novel profile of temperature dependence of Na^+^-K^+^ leak in a xerocytic form. Br. J. Haematol. 125, 521–527. 10.1111/j.1365-2141.2004.04944.x 15142123

[B58] Grootenboer S. Schischmanoff P. O. Laurendeau I. Cynober T. Tchernia G. Dommergues J. P.. (2000). Pleiotropic syndrome of dehydrated hereditary stomatocytosis, pseudohyperkalemia, and perinatal edema maps to 16q23-q24. Blood 96, 2599–2605. 11001917

[B59] Guizouarn H. Borgese F. Gabillat N. Harrison P. Goede J. S. McMahon C.. (2011). South-east Asian ovalocytosis and the cryohydrocytosis form of hereditary stomatocytosis show virtually indistinguishable cation permeability defects. Br. J. Haematol. 152, 655–664. 10.1111/j.1365-2141.2010.08454.x 21255002

[B60] Guizouarn H. Martial S. Gabillat N. Borgese F. (2007). Point mutations involved in red cell stomatocytosis convert the electroneutral anion exchanger 1 to a nonselective cation conductance. Blood 110, 2158–2165. 10.1182/blood-2006-12-063420 17554061

[B61] Hadley T. Saul A. Lamont G. Hudson D. E. Miller L. H. Kidson C. (1983). Resistance of Melanesian elliptocytes (ovalocytes) to invasion by Plasmodium knowlesi and *Plasmodium falciparum* malaria parasites *in vitro*. J. Clin. Invest. 71, 780–782. 10.1172/JCI110827 6338046 PMC436930

[B62] Haines P. G. Crawley C. Chetty M. C. Jarvis H. Coles S. E. Fisher J.. (2001b). Familial pseudohyperkalaemia Chiswick: a novel congenital thermotropic variant of K and Na transport across the human red cell membrane. Br. J. Haematol. 112, 469–474. 11167849 10.1046/j.1365-2141.2001.02564.x

[B63] Haines P. G. Jarvis H. G. King S. Noormohamed F. H. Chetty M. C. Fisher J.. (2001a). Two further British families with the “cryohydrocytosis” form of hereditary stomatocytosis. Br. J. Haematol. 113, 932–937. 11442486 10.1046/j.1365-2141.2001.02792.x

[B64] Hall A. C. Willis J. S. (1986). The temperature dependence of passive potassium permeability in mammalian erythrocytes. Cryobiology 23, 395–405. 10.1016/0011-2240(86)90024-6 3533429

[B65] Harm W. Fortier N. L. Lutz H. U. Fairbanks G. Snyder L. M. (1979). Increased erythrocyte lipid peroxidation in hereditary xerocytosis. Clin. Chim. Acta 99, 121–128. 10.1016/0009-8981(79)90034-2 509735

[B66] Helias V. Saison C. Ballif B. A. Peyrard T. Takahashi J. Takahashi H.. (2012). ABCB6 is dispensable for erythropoiesis and specifies the new blood group system Langereis. Nat. Genet. 44, 170–173. 10.1038/ng.1069 22246506 PMC3664204

[B67] Hoffman J. F. Joiner W. Nehrke K. Potapova O. Foye K. Wickrema A. (2003). The hSK4 (KCNN4) isoform is the Ca2+-activated K+ channel (Gardos channel) in human red blood cells. Proc. Natl. Acad. Sci. U.S.A. 100, 7366–7371. 10.1073/pnas.1232342100 12773623 PMC165881

[B68] Houston B. L. Zelinski T. Israels S. J. Coghlan G. Chodirker B. N. Gallagher P. G.. (2011). Refinement of the hereditary xerocytosis locus on chromosome 16q in a large Canadian kindred. Blood Cells Mol. Dis. 47, 226–231. 10.1016/j.bcmd.2011.08.001 21944700

[B69] Imashuku S. Muramatsu H. Sugihara T. Okuno Y. Wang X. Yoshida K.. (2016). PIEZO1 gene mutation in a Japanese family with hereditary high phosphatidylcholine hemolytic anemia and hemochromatosis-induced diabetes mellitus. Int. J. Hematol. 104, 125–129. 10.1007/s12185-016-1970-x 26971963

[B70] Iolascon A. De Falco L. Borgese F. Esposito M. R. Avvisati R. A. Izzo P.. (2009). A novel erythroid anion exchange variant (Gly796Arg) of hereditary stomatocytosis associated with dyserythropoiesis. Haematologica 94, 1049–1059. 10.3324/haematol.2008.002873 19644137 PMC2719027

[B71] Iolascon A. Stewart G. W. Ajetunmobi J. F. Perrotta S. Delaunay J. Carella M.. (1999). Familial pseudohyperkalemia maps to the same locus as dehydrated hereditary stomatocytosis (hereditary xerocytosis). Blood 93, 3120–3123. 10216110

[B72] Jarvis H. G. Chetty M. C. Nicolaou A. Fisher J. Miller A. Stewart G. W. (2001). A novel stomatocytosis variant showing marked abnormalities in intracellular [Na] and [K] with minimal haemolysis. Eur. J. Haematol. 66, 412–414. 10.1034/j.1600-0609.2001.066006412.x 11488942

[B73] Khan A. A. Hanada T. Mohseni M. Jeong J. J. Zeng L. Gaetani M.. (2008). Dematin and adducin provide a novel link between the spectrin cytoskeleton and human erythrocyte membrane by directly interacting with glucose transporter-1. J. Biol. Chem. 283, 14600–14609. 10.1074/jbc.M707818200 18347014 PMC2386908

[B74] Kiss K. Brozik A. Kucsma N. Toth A. Gera M. Berry L.. (2012). Shifting the paradigm: the putative mitochondrial protein ABCB6 resides in the lysosomes of cells and in the plasma membrane of erythrocytes. PLoS ONE 7:e37378. 10.1371/journal.pone.0037378 22655043 PMC3360040

[B75] Komatsu T. Sato K. Otsuka Y. Arashiki N. Tanaka K. Tamahara S.. (2010). Parallel reductions in stomatin and Na,K-ATPase through the exosomal pathway during reticulocyte maturation in dogs: stomatin as a genotypic and phenotypic marker of high K^+^ and low K^+^ red cells. J. Vet. Med. Sci. 72, 893–901. 10.1292/jvms.10-0030 20215716

[B76] Kucherenko Y. V. Huber S. M. Nielsen S. Lang F. (2012). Decreased redox-sensitive erythrocyte cation channel activity in aquaporin 9-deficient mice. J. Membr. Biol. 245, 797–805. 10.1007/s00232-012-9482-y 22836670

[B77] Kuma H. Abe Y. Askin D. Bruce L. J. Hamasaki T. Tanner M. J.. (2002). Molecular basis and functional consequences of the dominant effects of the mutant band 3 on the structure of normal band 3 in Southeast Asian ovalocytosis. Biochemistry 41, 3311–3320. 10.1021/bi011678 11876639

[B78] Lande W. M. Thiemann P. V. Mentzer W. C. Jr. (1982). Missing band 7 membrane protein in two patients with high Na, low K erythrocytes. J. Clin. Invest. 70, 1273–1280. 10.1172/JCI110726 7174793 PMC370344

[B79] Lauf P. K. Adragna N. C. (2000). K-Cl cotransport: properties and molecular mechanism. Cell. Physiol. Biochem. 10, 341–354. 10.1159/000016357 11125215

[B80] Lauf P. K. Joiner C. H. (1976). Increased potassium transport and ouabain binding in human Rhnull red blood cells. Blood 48, 457–468. 133741

[B81] Lim J. Gasson C. Kaji D. M. (1995). Urea inhibits NaK2Cl cotransport in human erythrocytes. J. Clin. Invest. 96, 2126–2132. 10.1172/JCI118266 7593597 PMC185861

[B82] Lock S. P. Smith R. S. Hardisty R. M. (1961). Stomatocytosis: a hereditary red cell anomally associated with haemolytic anaemia. Br. J. Haematol. 7, 303–314. 10.1111/j.1365-2141.1961.tb00341.x 13762977

[B83] Lytle C. McManus T. (2002). Coordinate modulation of Na-K-2Cl cotransport and K-Cl cotransport by cell volume and chloride. Am. J. Physiol. Cell Physiol. 283, C1422–C1431. 10.1152/ajpcell.00130.2002 12372803

[B84] Marini A. M. Urrestarazu A. Beauwens R. André B. (1997). The Rh (rhesus) blood group polypeptides are related to NH4^+^ transporters. Trends Biochem. Sci. 22, 460–461. 10.1016/S0968-0004(97)01132-8 9433124

[B85] Meadow S. R. (1967). Stomatocytosis. Proc. R. Soc. Med. 60, 13–15. 6018468 10.1177/003591576706000108 PMC1901440

[B86] Meenaghan M. Follett G. F. Brophy P. J. (1985). Temperature sensitivity of potassium flux into red blood cells in the familial pseudohyperkalaemia syndrome. Biochim. Biophys. Acta 821, 72–78. 10.1016/0005-2736(85)90155-5 2998465

[B87] Miller D. R. Rickles F. R. Lichtman M. A. La Celle P. L. Bates J. Weed R. I. (1971). A new variant of hereditary hemolytic anemia with stomatocytosis and erythrocyte cation abnormality. Blood 38, 184–204. 5559828

[B88] Montel-Hagen A. Kinet S. Manel N. Mongellaz C. Prohaska R. Battini J. L.. (2008). Erythrocyte Glut1 triggers dehydroascorbic acid uptake in mammals unable to synthesize vitamin C. Cell 132, 1039–1048. 10.1016/j.cell.2008.01.042 18358815

[B89] Morlé L. Pothier B. Alloisio N. Féo C. Garay R. Bost M.. (1989). Reduction of membrane band 7 and activation of volume stimulated (K+, Cl-)-cotransport in a case of congenital stomatocytosis. Br. J. Haematol. 71, 141–146. 10.1111/j.1365-2141.1989.tb06288.x 2917122

[B90] Nunomura W. Denker S. P. Barber D. L. Takakuwa Y. Gascard P. (2012). Characterization of cytoskeletal protein 4.1R interaction with NHE1 (Na(+)/H(+) exchanger isoform 1). Biochem. J. 446, 427–435. 10.1042/BJ20120535 22731252 PMC4338608

[B91] Paessler M. Hartung H. (2015). Dehydrated hereditary stomatocytosis masquerading as MDS. Blood 125:1841. 25927085 10.1182/blood-2014-11-612184

[B92] Palsdottir H. Hunte C. (2004). Lipids in membrane protein structures. Biochim. Biophys. Acta 1666, 2–18. 10.1016/j.bbamem.2004.06.012 15519305

[B93] Parker J. C. McManus T. J. Starke L. C. Gitelman H. J. (1990). Coordinated regulation of Na/H exchange and [K-Cl] cotransport in dog red cells. J. Gen. Physiol. 96, 1141–1152. 10.1085/jgp.96.6.1141 1962814 PMC2229031

[B94] Platt O. S. Lux S. E. Nathan D. G. (1981). Exercise-induced hemolysis in xerocytosis. Erythrocyte dehydration and shear sensitivity. J. Clin. Invest. 68, 631–638. 10.1172/JCI110297 7276163 PMC370843

[B95] Ranade S. S. Qiu Z. Woo S. H. Hur S. S. Murthy S. E. Cahalan S. M.. (2014). Piezo1, a mechanically activated ion channel, is required for vascular development in mice. Proc. Natl. Acad. Sci. U.S.A. 111, 10347–10352. 10.1073/pnas.1409233111 24958852 PMC4104881

[B96] Rapetti-Mauss R. Lacoste C. Picard V. Guitton C. Pontou-Lombard E. Loosveld M.. (2015). A mutation in the Gardos channel is associated with hereditary xerocytosis. Blood 126, 1273–1280. 10.1182/blood-2015-04-642496 26148990

[B97] Rapetti-Mauss R. Picard V. Guitton C. Ghazal K. Proulle V. Badens C.. (2017). Red blood cell Gardos channel (KCNN4): the essential determinant of erythrocyte dehydration in hereditary xerocytosis. Haematologica 102, e415–e418. 10.3324/haematol.2017.171389 28619848 PMC5622875

[B98] Rees D. C. Iolascon A. Carella M. O'marcaigh A. S. Kendra J. R. Jowitt S. N.. (2005). Stomatocytic haemolysis and macrothrombocytopenia (Mediterranean stomatocytosis/macrothrombocytopenia) is the haematological presentation of phytosterolaemia. Br. J. Haematol. 130, 297–309. 10.1111/j.1365-2141.2005.05599.x 16029460

[B99] Reithmeier R. A. Casey J. R. Kalli A. C. Sansom M. S. Alguel Y. Iwata S. (2016). Band 3, the human red cell chloride/bicarbonate anion exchanger (AE1, SLC4A1), in a structural context. Biochim. Biophys. Acta 1858(7 Pt A):1507–1532. 10.1016/j.bbamem.2016.03.030 27058983

[B100] Rigor R. R. Damoc C. Phinney B. S. Cala P. M. (2011). Phosphorylation and activation of the plasma membrane Na+/H+ exchanger (NHE1) during osmotic cell shrinkage. PLoS ONE 6:e29210. 10.1371/journal.pone.0029210 22216214 PMC3247252

[B101] Rivera A. Kam S. Y. Ho M. Romero J. R. Lee S. (2013a). Ablation of the Kell/Xk complex alters erythrocyte divalent cation homeostasis. Blood Cells Mol. Dis. 50, 80–85. 10.1016/j.bcmd.2012.10.002 23122227 PMC3540154

[B102] Rivera A. Zee R. Y. Alper S. L. Peters L. L. Brugnara C. (2013b). Strain-specific variations in cation content and transport in mouse erythrocytes. Physiol. Genomics 45, 343–350. 10.1152/physiolgenomics.00143.2012 23482811 PMC3656420

[B103] Rotstein M. Engelstad K. Yang H. Wang D. Levy B. Chung W. K.. (2010). Glut1 deficiency: inheritance pattern determined by haploinsufficiency. Ann. Neurol. 68, 955–958. 10.1002/ana.22088 20687207 PMC2994988

[B104] Rungaldier S. Oberwagner W. Salzer U. Csaszar E. Prohaska R. (2013). Stomatin interacts with GLUT1/SLC2A1, band 3/SLC4A1, and aquaporin-1 in human erythrocyte membrane domains. Biochim. Biophys. Acta 1828, 956–966. 10.1016/j.bbamem.2012.11.030 23219802 PMC3790964

[B105] Salzer U. Prohaska R. (2001). Stomatin, flotillin-1, and flotillin-2 are major integral proteins of erythrocyte lipid rafts. Blood 97, 1141–1143. 10.1182/blood.V97.4.1141 11159550

[B106] Sandberg M. B. Nybo M. Birgens H. Frederiksen H. (2014). Hereditary xerocytosis and familial haemolysis due to mutation in the PIEZO1 gene: a simple diagnostic approach. Int. J. Lab. Hematol. 36, e62–e65. 10.1111/ijlh.12172 24314002

[B107] Shmukler B. E. Vandorpe D. H. Rivera A. Auerbach M. Brugnara C. Alper S. L. (2014). Dehydrated stomatocytic anemia due to the heterozygous mutation R2456H in the mechanosensitive cation channel PIEZO1: a case report. Blood Cells Mol. Dis. 52, 53–54. 10.1016/j.bcmd.2013.07.015 23973043

[B108] Stewart A. K. Kedar P. S. Shmukler B. E. Vandorpe D. H. Hsu A. Glader B.. (2011). Functional characterization and modified rescue of novel AE1 mutation R730C associated with overhydrated cation leak stomatocytosis. Am. J. Physiol. Cell Physiol. 300, C1034–C1046. 10.1152/ajpcell.00447.2010 21209359 PMC3093938

[B109] Stewart A. K. Vandorpe D. H. Heneghan J. F. Chebib F. Stolpe K. Akhavein A.. (2010). The GPA-dependent, spherostomatocytosis mutant AE1 E758K induces GPA-independent, endogenous cation transport in amphibian oocytes. Am. J. Physiol. Cell Physiol. 298, C283–C297. 10.1152/ajpcell.00444.2009 19907019 PMC2822494

[B110] Stewart G. W. (2004). Hemolytic disease due to membrane ion channel disorders. Curr. Opin. Hematol. 11, 244–250. 10.1097/01.moh.0000132240.20671.33 15314523

[B111] Stewart G. W. Amess J. A. Eber S. W. Kingswood C. Lane P. A. Smith B. D.. (1996). Thrombo-embolic disease after splenectomy for hereditary stomatocytosis. Br. J. Haematol. 93, 303–310. 10.1046/j.1365-2141.1996.4881033.x 8639421

[B112] Stewart G. W. Corrall R. J. Fyffe J. A. Stockdill G. Strong J. A. (1979). Familial pseudohyperkalaemia. A new syndrome. Lancet 2, 175–177. 89283 10.1016/s0140-6736(79)91437-5

[B113] Stewart G. W. Ellory J. C. (1985). A family with mild hereditary xerocytosis showing high membrane cation permeability at low temperatures. Clin. Sci. 69, 309–319. 10.1042/cs0690309 4064573

[B114] Stewart G. W. Ellory J. C. Klein R. A. (1980). Increased human red cell cation passive permeability below 12 degrees C. Nature 286, 403–404. 10.1038/286403a0 7402324

[B115] Stewart G. W. Hepworth-Jones B. E. Keen J. N. Dash B. C. Argent A. C. Casimir C. M. (1992). Isolation of cDNA coding for an ubiquitous membrane protein deficient in high Na+, low K+ stomatocytic erythrocytes. Blood 79, 1593–1601. 1547348

[B116] Syeda R. Xu J. Dubin A. E. Coste B. Mathur J. Huynh T.. (2015). Chemical activation of the mechanotransduction channel Piezo1. Elife 4. 10.7554/eLife.07369 26001275 PMC4456433

[B117] Syfuss P. Y. Ciupea A. Brahimi S. Cynober T. Stewart G. W. Grandchamp B.. (2006). Mild dehydrated hereditary stomatocytosis revealed by marked hepatosiderosis. Clin. Lab. Haematol. 28, 270–274. 10.1111/j.1365-2257.2006.00774.x 16898969

[B118] Tanner M. J. Bruce L. Martin P. G. Rearden D. M. Jones G. L. (1991). Melanesian hereditary ovalocytes have a deletion in red cell band 3. Blood 78, 2785–2786. 1824272

[B119] Weber Y. G. Storch A. Wuttke T. V. Brockmann K. Kempfle J. Maljevic S.. (2008). GLUT1 mutations are a cause of paroxysmal exertion-induced dyskinesias and induce hemolytic anemia by a cation leak. J. Clin. Invest. 118, 2157–2168. 10.1172/JCI34438 18451999 PMC2350432

[B120] Wiley J. S. (1981). Increased erythrocyte cation permeability in thalassemia and conditions of marrow stress. J. Clin. Invest. 67, 917–922. 10.1172/JCI110140 7204577 PMC370647

[B121] Zarychanski R. Schulz V. P. Houston B. L. Maksimova Y. Houston D. S. Smith B.. (2012). Mutations in the mechanotransduction protein PIEZO1 are associated with hereditary xerocytosis. Blood 120, 1908–1915. 10.1182/blood-2012-04-422253 22529292 PMC3448561

